# Dietary Sodium Restriction and Frailty among Middle-Aged and Older Adults: An 8-Year Longitudinal Study

**DOI:** 10.3390/nu16050580

**Published:** 2024-02-20

**Authors:** Yu-Chun Lin, Huang-Ting Yan

**Affiliations:** 1 Department of Chinese Medicine, China Medical University Hospital, Taichung City 40447, Taiwan; yclinjoyce@gmail.com; 2 Graduate Institute of Integrated Medicine, China Medical University, Taichung City 40402, Taiwan; 3 Institute of Political Science, Academia Sinica, Taipei City 11529, Taiwan

**Keywords:** dietary sodium restriction, frailty, frailty phenotypes, older adults

## Abstract

Frailty is a common geriatric syndrome. However, there is little information about the relationship between dietary sodium restriction (DSR) and frailty in later life. This study aimed to elucidate the relationship between DSR and frailty in middle-aged and older adults. The 8-year follow-up data from the Taiwan Longitudinal Study on Aging, including 5131 individuals aged ≥50 years, were analyzed using random-effects panel logit models. DSR was evaluated by assessing whether the participants were told by a physician to reduce or avoid sodium intake from food. Three indices were used to measure frailty: the Study of Osteoporotic Fractures (SOF) index, the Fried index, and the Fatigue, Resistance, Ambulation, Illness, and Loss of weight (FRAIL) index. Individuals with DSR were more likely to report frailty compared with those with non-DSR (SOF: adjusted odds ratio [AOR] = 1.82, 95% confidence interval [CI] = 1.46–2.27; Fried: AOR = 2.55, 95% CI = 1.64–3.98; FRAIL: AOR = 2.66, 95% CI = 1.89–3.74). DSR was associated with a higher likelihood of SBF (AOR = 2.61, 95% CI = 1.61–4.22). We identified a temporal trajectory in our study, noting significant participant reactions to both short- and mid-term DSR. Future research should address the balance between frailty risk and cardiovascular risk related to DSR.

## 1. Introduction

Frailty is a common geriatric syndrome characterized by an age-dependent decline in the functioning of multiple organ systems, leading to the loss of biological reserves, elevated vulnerability to stressors [1], and a higher risk of adverse health outcomes, including loss of activities of daily living, falls and fractures, hospitalization, and mortality [2]. In previous population-based studies from 62 countries, the global prevalence of frailty among people aged ≥50 years is estimated to range from 12% to 24%, as determined by various frailty scales [3].

A broad body of literature has confirmed that poor nutritional status in old age is associated with increased frailty [4,5,6,7,8,9,10]. A previous cross-sectional study involving 1200 community-dwelling rural Lebanese individuals aged 65 years or older found a strong and independent relationship between frailty, malnutrition, and the risk of malnutrition [4]. Similarly, several cross-sectional studies conducted on community-dwelling older adults from Germany [5], Malaysia [6], Singapore [7], South Korea [8], and Taiwan [9] indicated that malnutrition and its risk were significant predictors of pre-frailty and frailty. Furthermore, a meta-analysis supported the association between physical frailty, sarcopenia, and malnutrition in hospitalized older adults [10]. Numerous studies have contributed to the body of evidence highlighting the correlation between nutrients and frailty, confirming that frailty is independently associated with a low intake of specific micronutrients [11,12,13] and macronutrients [14,15,16].

Dietary sodium restriction (DSR) is a widely recommended self-care behavior for patients with heart failure [17] and kidney disease [18]. Despite its common prescription, DSR may have limited advantages [19] and potentially result in adverse health outcomes [20]. Evidence suggests that DSR does not improve the long-term quality of life and may increase the risk of mortality and readmission rate in individuals with various primary heart diseases [20]. In patients with heart failure, elevated plasma aldosterone levels resulting from salt restriction trigger mineralocorticoid receptor signaling under volume-overloaded conditions, resulting in increased myocardial fibrosis [21]. Furthermore, salt restriction is associated with low caloric intake and malnutrition, particularly in older patients [22]. Recent research has revealed a relationship between DSR and falling experiences in middle-aged and older adults, suggesting a potential pathway linking DSR, impaired nutritional status, decreased functional capacity, and falls in older adults [23]. Considering the close association of nutritional status and diminished functional capacity with frailty, this study argues that a low dietary sodium intake may contribute to frailty in older adults.

This study employs panel data covering 5131 individuals aged 50 years or older in three waves from 1999 to 2007 to investigate the relationship between DSR and frailty.

## 2. Materials and Methods

### 2.1. Study Population

The unit of analysis was “individuals-year”. This study extracted data collected from the Taiwan Longitudinal Study on Aging (TLSA). It was a nationally representative cohort study that included the adult population aged 50 years or older. A three-stage equal-probability sampling was used for the selection of a nationally representative sample from household registration data. The data were collected through face-to-face interview questionnaires across six waves from 1989 to 2007. We selected three of the six survey rounds, 1999, 2003, and 2007, for analysis of a sample of pooled cross-sectional and time-series observations.

### 2.2. Study Variables

The DSR was assessed by asking whether the participants were specifically instructed by a physician to reduce or avoid sodium intake from food due to illness. The DSR data were collected in 1999 as explanatory variables. A dummy variable was created, with 1 indicating those who responded “yes” and 0 indicating those who responded otherwise.

Several indices have been developed to assess frailty. This study applied the Study of Osteoporotic Fractures (SOF) index [24], the Cardiovascular Health Study (CHS) index (also known as the Fried index) [25], and the Fatigue, Resistance, Ambulation, Illness, and Loss of weight (FRAIL) index [26]. We made adjustments to specific assessment questions. The SOF index includes three components: weight loss (the involuntary loss of 3 kg of body weight in the past year), chair stands (difficulty squatting), and reduced energy level (self-perceived reduced energy level as described by an answer of “yes, often, or chronically” to the question “In the past week, do you feel that you have been unable to gather your energy to do things?”). Participants were considered “frail” if at least two of the three criteria were met.

The Fried index consists of five elements: weight loss, fatigue/exhaustion, weakness, slowness, and low physical activity. Weight loss and fatigue/exhaustion were evaluated using identical criteria, which were defined as weight loss and reduced energy level in the SOF index. Weakness was determined by asking the participants if they experienced difficulties using their fingers to grasp or turn objects. Slowness was evaluated by asking participants if they faced challenges walking 200–300 m. Low physical activity was assessed based on the incidence and progression of basic activities of daily living disability, derived from modified activities of daily living (ADL) index. The following question was used: “I will mention some common daily activities. Please tell me if you have any difficulty doing these by yourself (bathing, dressing, undressing, feeding, getting out of bed, standing up, sitting on a chair, walking, and toileting)”. Participants who encountered difficulties in performing at least one of these activities were considered physically inactive. Those who met three or more of these criteria were considered “frail”.

The FRAIL index consists of five components, each designated by the initial letter of its primary name. These components included fatigue, resistance, ambulation, illness, and weight loss. Fatigue was measured by asking participants whether they experienced reduced energy levels to perform any task in the past week, with a “yes, often, or chronic response” obtaining a score of 1 point. Resistance was determined by asking the participants if they encountered any difficulty walking up two or three flights of stairs without using aids, with a “yes” response obtaining a score of 1 point. Ambulation was evaluated by asking the participants if they had any difficulty walking 200–300 m, with a “yes” response obtaining a score of 1 point. Illness was assigned a score of 1 point if the participant reported taking medications or receiving treatment for four or more of the following chronic diseases: hypertension, diabetes, heart disease, stroke, cancer, lung disease, arthritis, or renal disease. Weight loss was identified by asking the participants if they had lost more than 3 kg of body weight in the previous year, with a “yes” response obtaining a score of 1 point. Scores of 3–5 indicate a “frail” health status. Data were collected in 1999, 2003, and 2007.

Two frailty phenotypes were identified in this study. The energy-based frailty (EBF) index has two components: losing weight and fatigue. Participants were considered “energy-based frail” if they fulfilled two of the EBF criteria. The sarcopenia-based frailty (SBF) index has four components: low handgrip strength (inability to use fingers to grasp or turn objects), low walking ability (inability to walk 200–300 m), low resistance (inability to squat), and low physical activity (inability to perform some common daily activities). The participants were considered “sarcopenia-based frail” if they fulfilled at least three of the four SBF criteria. Older adults with energy- and sarcopenia-based frailties were classified as having hybrid-based frailty.

Individual-level characteristics were included as covariates. This included age, sex (binary, 1 = male), educational attainment level (0 years, 1–6 years, 7–12 years, and ≥13 years), current living status (binary, 1 = living alone), marital status (binary, 1 = married or living with a partner), alcohol intake (binary, 1 = alcohol drinker), smoking status (binary, 1 = smoker), and exercise frequency (0, 1, 2, and ≥ 3 times per week). Table 1 presents the characteristics of the sample (frailty status defined based on the SOF index). The sample size was 3593, 2669, and 2095, according to the different waves in Table 1.

### 2.3. Statistical Analysis

To describe the study data, descriptive statistics were used, including absolute and percentage frequency distributions. The Chi-square and one-way analysis of variance (ANOVA) were used to compare categorical and continuous variables, respectively, with *p* < 0.05 indicating statistical significance.

The study used the panel data of 5131 people aged ≥50 years from 1999 to 2007, yielding a sample of pooled cross-sectional and time-series observations. Several fixed-effects and random-effects models can be employed with panel data depending on various factors. The standard errors generated by the fixed-effects models might be too large to tolerate if there is a limited degree of variability among the participants, as these models require within-subject variations in the variables. In such cases, random-effects models prove to be more suitable. The majority of older adults exhibited minimal change over time; therefore, this study used a random-effects panel logit model. To identify a temporal trajectory of how DSR is associated with frailty and their respective phenotypes, in comparison to non-DSR, we computed the interaction term: “DSR × years”. Three interaction terms, “DSR × sex (binary, 1 = male)”, “DSR × educational attainment level (binary, 1 = formal education)”, and “DSR × age groups (binary, 1 = 80 years and above)”, were used to determine the subgroups under which DSR exerts an impact on the likelihood of frailty. The Stata software was used for statistical analyses.

### 2.4. Robustness Tests

For the robustness tests, the outcomes were categorized into three groups: frail, pre-frail (SOF: only one criterion; Fried: one or two criteria; FRAIL: scores of 1–2; EBF: only one criterion; SBF: one or two criteria), and robust. Second, the SBF scale primarily assesses the upper (low handgrip strength) and lower limb muscle strength (low resistance), physical function (low walking ability), and physical performance (low physical activity). This study used an alternative method for measuring lower limb muscle strength by asking participants if they had any difficulty walking up two or three flights of stairs without using aids. Third, the study excluded individuals diagnosed with hypertension or chronic kidney disease and prescribed medications by a physician in 1999; these specific characteristics may synchronously lead to DSR, which is required by a doctor to control hypertension or chronic kidney disease [17,18], and a greater likelihood of frailty.

## 3. Results

**Figure 1:**
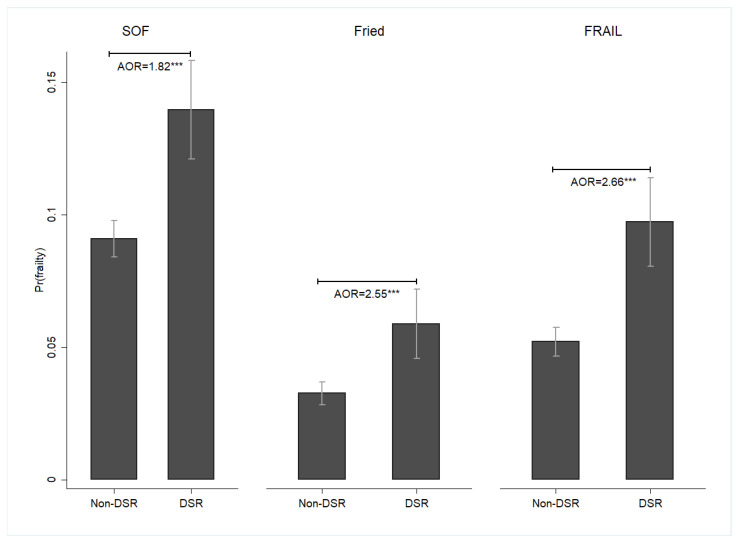
The effects of DSR on the probability of frailty among middle-aged and older adults, Taiwan, 1999–2007. Note: All results were obtained using a random-effects panel logit model. The mean and standard error were represented by bar charts with error bars. Pr(frailty) was used to represent the predicted probability of frailty. DSR: dietary sodium restriction; AOR: adjusted odds ratio. Pr(frailty) (SOF): DSR: 0.14 ***, CI = 0.12–0.16; Non-DSR: 0.09 ***, CI = 0.08–0.10; Pr(frailty) (Fried): DSR: 0.06 ***, CI = 0.05–0.07; Non-DSR: 0.03 ***, CI = 0.03–0.04; Pr(frailty) (FRAIL): DSR: 0.10 ***, CI = 0.08–0.11; Non-DSR: 0.05 ***, CI = 0.05–0.06; *** *p* < 0.001. Source: the author.

**Figure 2:**
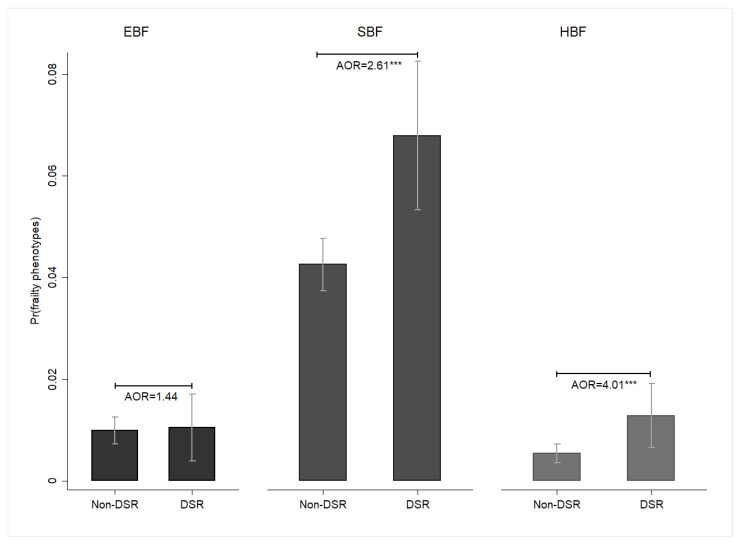
The effects of DSR on the probability of frailty phenotypes among middle-aged and older adults, Taiwan, 1999–2007. Note: All results were obtained using a random-effects panel logit model. The mean and standard error were represented by bar charts with error bars. Pr(frailty phenotypes) was used to represent the predicted probability of frailty phenotypes. DSR: dietary sodium restriction; EBF: energy-based frailty; SBF: sarcopenia-based frailty; HBF: hybrid-based frailty; AOR: adjusted odds ratio. Pr(frailty phenotypes) (EBF): DSR: 0.01 **, CI = 0.004–0.02; Non-DSR: 0.01 ***, CI = 0.01–0.01; Pr(frailty phenotypes) (SBF): DSR: 0.07 ***, CI = 0.05–0.08; Non-DSR: 0.04 ***, CI = 0.04–0.05; Pr(frailty phenotypes) (HBF): DSR: 0.01 ***, CI = 0.01–0.02; Non-DSR: 0.01 ***, CI = 0.004–0.01; *** *p* < 0.001. Source: the author.

The DSR group was more likely to report frailty compared with the non-DSR group (SOF: adjusted odds ratio [AOR] = 1.82, 95% confidence interval [CI] = 1.46–2.27; Fried: AOR = 2.55, 95% CI = 1.64–3.98; FRAIL: AOR = 2.66, 95% CI = 1.89–3.74) (Figure 1). Furthermore, DSR was associated with a higher likelihood of SBF (AOR = 2.61, 95% CI = 1.61–4.22). However, no significant differences were observed in the likelihood of EBF between the DSR and non-DSR groups (AOR = 1.44, 95% CI = 0.66–3.15) (Figure 2).

**Figure 3:**
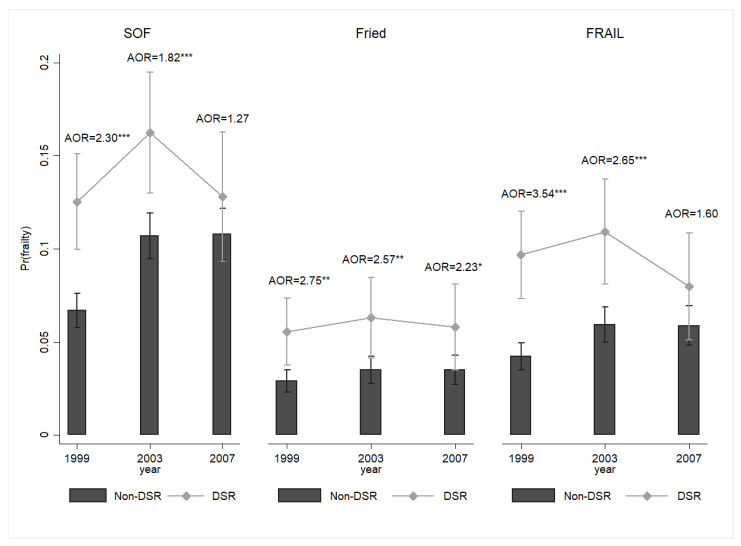
The temporal effects of DSR on the probability of frailty among middle-aged and older adults, Taiwan, 1999–2007. Note: All results were obtained using a random-effects panel logit model. The mean and standard error were represented by bar charts with error bars. Pr(frailty) was used to represent the predicted probability of frailty. DSR: dietary sodium restriction; AOR: adjusted odds ratio. Pr(frailty) (SOF): DSR, 1999: 0.13***, CI = 0.10–0.15; Non-DSR, 1999: 0.07 ***, CI = 0.06–0.08; DSR, 2003: 0.16 ***, CI = 0.13–0.19; Non-DSR, 2003: 0.11 ***, CI = 0.09–0.12; DSR, 2007: 0.13 ***, CI = 0.09–0.16; Non-DSR, 2007: 0.11 ***, CI = 0.09–0.12; Pr(frailty) (Fried): DSR, 1999: 0.06 ***, CI = 0.04–0.07; Non-DSR, 1999: 0.03 ***, CI = 0.02–0.04; DSR, 2003: 0.06 ***, CI = 0.04–0.08; Non-DSR, 2003: 0.04 ***, CI = 0.03–0.04; DSR, 2007: 0.06 ***, CI = 0.04–0.08; Non-DSR, 2007: 0.04 ***, CI = 0.03–0.04; Pr(frailty) (FRAIL): DSR, 1999: 0.10 ***, CI = 0.07–0.12; Non-DSR, 1999: 0.04 ***, CI = 0.03–0.05; DSR, 2003: 0.11 ***, CI = 0.08–0.14; Non-DSR, 2003: 0.06 ***, CI = 0.05–0.07; DSR, 2007: 0.08 ***, CI = 0.05–0.11; Non-DSR, 2007: 0.06 ***, CI = 0.05–0.07; * *p* < 0.05, ** *p* < 0.01, and *** *p* < 0.001. Source: the author.

**Figure 4:**
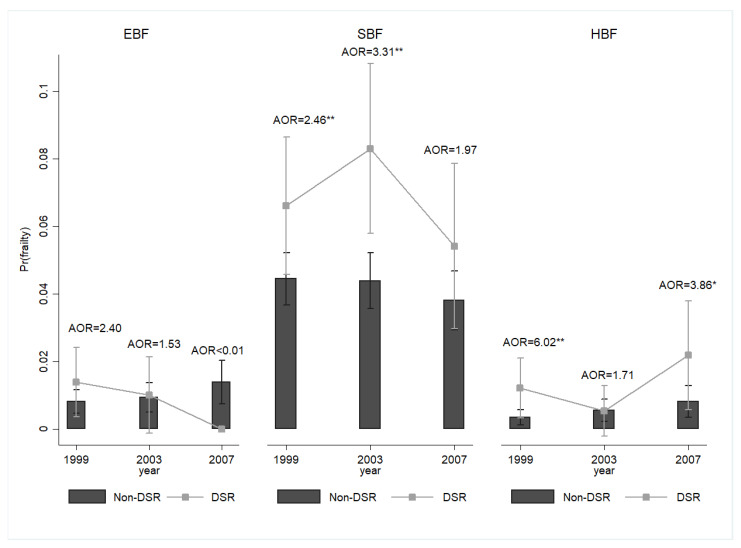
The temporal effects of DSR on the probability of frailty phenotypes among middle-aged and older adults, Taiwan, 1999–2007. Note: All results were obtained using a random-effects panel logit model. The mean and standard error were represented by bar charts with error bars. Pr(frailty phenotypes) was used to represent the predicted probability of frailty phenotypes. DSR: dietary sodium restriction; EBF: energy-based frailty; SBF: sarcopenia-based frailty; HBF: hybrid-based frailty; AOR: adjusted odds ratio. Pr(frailty phenotypes) (SBF): DSR, 1999: 0.07 ***, CI = 0.05–0.09; Non-DSR, 1999: 0.04 ***, CI = 0.04–0.05; DSR, 2003: 0.08 ***, CI = 0.06–0.11; Non-DSR, 2003: 0.04 ***, CI = 0.04–0.05; DSR, 2007: 0.05 ***, CI = 0.03–0.08; Non-DSR, 2007: 0.04 ***, CI = 0.03–0.05; * *p* < 0.05, ** *p* < 0.01. Source: the author.

A temporal effect may have occurred (Figure 3). Compared with the non-DSR group, the DSR group was more likely to be frail in 1999 (AOR = 2.30, 95% CI = 1.64–3.22) and 2003 (AOR = 1.82, 95% CI = 1.29–2.57). However, no significant difference was found in the likelihood of frailty between the DSR and non-DSR groups in 2007 (AOR = 1.27, 95% CI = 0.84–1.94). When the frailty diagnosis was made based on the Fried (1999: AOR = 2.75, 95% CI = 1.48–5.12; 2003: AOR = 2.57, 95% CI = 1.30–5.05; 2007: AOR = 2.23, 95% CI = 1.05–4.73) or FRAIL (1999: AOR = 3.54, 95% CI = 2.18–5.76; 2003: AOR = 2.65, 95% CI = 1.59–4.44; 2007: AOR = 1.60, 95% CI = 0.86–3.00) criteria, the pattern was similar. We also found a temporal trajectory of the SBF likelihood: the participants’ reactions to short- and mid-term DSR were significant (1999: AOR = 2.46, 95% CI = 1.28–4.72; 2003: AOR = 3.31, 95% CI = 1.62–6.74; 2007: AOR = 1.97, 95% CI = 0.82–4.72). No temporal differences were observed in the likelihood of EBF (Figure 4).

**Figure 5:**
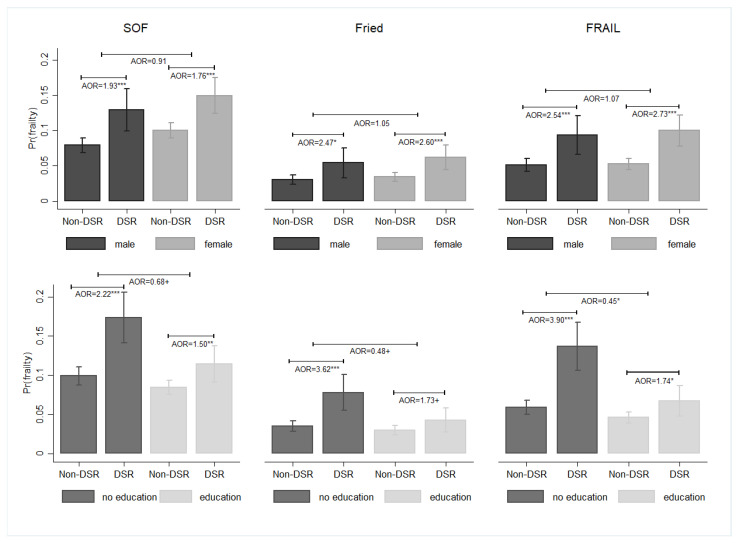
The subgroup effects of DSR on the probability of frailty among middle-aged and older adults, Taiwan, 1999–2007. Note: All results were obtained using a random-effects panel logit model. The mean and standard error were represented by bar charts with error bars. Pr(frailty) was used to represent the predicted probability of frailty. DSR: dietary sodium restriction; AOR: adjusted odds ratio. ^+^ *p* < 0.1, * *p* < 0.05, ** *p* < 0.01, and *** *p* < 0.001. Source: the author.

Men who were required to reduce sodium intake by a physician were more prone to frailty than their counterparts who did not receive DSR (SOF: AOR = 1.93, 95% CI = 1.35–2.77; Fried: AOR = 2.47, 95% CI = 1.22–5.01; FRAIL: AOR = 2.54, 95% CI = 1.48–4.36) (Figure 5). This phenomenon was also observed in women. However, no significant difference was found between male and female older adults in terms of the association between DSR and the likelihood of frailty. By contrast, the association of DSR with the likelihood of frailty in older adults was more prevalent in groups with no formal education than in groups with formal education (e.g., SOF: no formal education: AOR = 2.22, 95% CI = 1.61–3.06; formal education: AOR = 1.50, 95% CI = 1.11–2.05).

**Figure 6:**
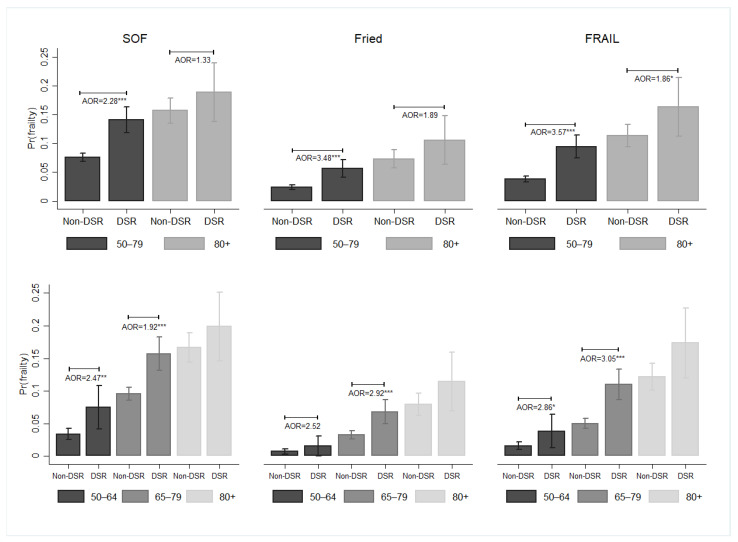
The subgroup effects of DSR on the probability of frailty among middle-aged and older adults, Taiwan, 1999–2007. Note: All results were obtained using a random-effects panel logit model. The mean and standard error were represented by bar charts with error bars. Pr(frailty) was used to represent the predicted probability of frailty. Participants were divided into two or three age groups. DSR: dietary sodium restriction; AOR: adjusted odds ratio. ^+^ *p* < 0.1, * *p* < 0.05, ** *p* < 0.01, and *** *p* < 0.001. Source: the author.

There was an age-dependent effect of how DSR was associated with frailty (Figure 6). When individuals spanned the age range of 50 to 79 years old, DSR was associated with a greater likelihood of frailty (SOF: AOR = 2.28, 95% CI = 1.76–2.94; Fried: AOR: 3.48, 95% CI: 2.09–5.80; FRAIL: AOR: 3.57, 95% CI: 2.42–5.27). This effect was not statistically significant for those aged 80 and above. The results were obtained by dividing individuals into three age groups.

The results of the robustness tests aligned with our primary analysis of the impact of DSR (Table S1). When the outcome variable was categorized into three distinct groups, DSR, compared with non-DSR, exhibited an increased likelihood of pre-frailty in contrast to the robust groups (SOF: AOR = 2.04, 95% CI = 1.69–2.46; Fried: AOR = 1.61, 95% CI = 1.38–1.87; FRAIL: AOR = 2.03, 95% CI = 1.71–2.42) (Figure S1). Alternatively, the non-DSR group was more likely to have a robust health status compared with the DSR group. Additionally, a temporal pattern was observed in the impact of DSR on the likelihood of pre-frailty (Figure S2).

## 4. Discussion

Individuals with DSR were more likely to report frailty than those without DSR. DSR may contribute to poor appetite. The lack of appetite in older adults is called anorexia of aging and is related to a decreased feeding drive [27]. This decline is attributed to the age-related deterioration in the ability to identify smell and taste, affecting the overall sensation of enjoying food and subsequently affecting nutritional intake [28]. Older patients are particularly susceptible to low sodium intake because the unappealing nature of low-sodium foods leads to a general decrease in protein and calorie intake, which may adversely affect their quality of life [29]. Recent studies have demonstrated the association of low daily sodium intake with an inadequate intake of calories, carbohydrates, proteins, and fiber, as well as a deficiency in vitamins and minerals [22,30,31]. The lack of nutritional intake is a crucial risk factor for frailty [4,5,6,7,8,9,10,11,12,13,14,15,16], as a decrease in nutritional intake results in a reduction in muscle mass and strength. Existing evidence suggests that frailty and pre-frailty are closely associated with appetite loss in older adults [32,33,34].

The DSR was associated with a significantly increased risk of SBF. Several studies have indicated that older adults with malnutrition had a significantly lower handgrip strength [35,36], low gait speed [35,37], difficulty performing a chair stand test [35,37], low muscle mass [35,36], and poor muscle health [38,39] compared with their counterparts with normal nutrition. In cases of malnutrition, the adipose tissue is the body’s main energy source for maximizing muscle preservation. When the body does not receive enough energy, amino acids stored as proteins in the muscle are broken down to provide the body with energy through gluconeogenesis. This process leads to muscle protein catabolism and a subsequent decline in body muscle mass [40]. Malnourished older adults also exhibit poor performance in daily living activities [41,42,43]; malnutrition is a leading cause of decreased muscle mass, resulting in diminished physical function. This may have a direct impact on the performance of predominantly physically demanding daily tasks [44]. The primary dimensions of the SBF scale are muscle strength and functional capacity. This finding suggests a potential pathway linking DSR, poor appetite, compromised nutritional status, and SBF in older adults.

No significant association was observed between DSR and the likelihood of EBF. One potential explanation is that DSR directly affects nutritional intake, muscle mass, and strength, subsequently leading to indirect consequences, such as body weight loss and fatigue. When older adults with EBF and SBF are categorized as having hybrid-based frailty, weight loss and fatigue, which comprise the EBF scale, may not be necessarily related to the subsequent changes in appetite caused by DSR. Indeed, fatigue cannot be definitively attributed to or entirely explained by a single illness in many older adults, and the underlying pathophysiological mechanisms remain unclear [45].

The observed phenomenon may not be attributed to the risk of DSR, but to the potentially hazardous condition that requires sodium restriction. Therefore, an instrumental variable is necessary to eliminate endogeneity. However, finding suitable instruments for assessing DSR poses a challenge, given that health variables correlated with DSR are highly likely to influence the likelihood of frailty. To avoid spurious interrelationships, individuals with risk conditions that led to DSR and a greater likelihood of frailty were excluded. We identified two risky conditions that warrant sodium restriction: chronic kidney disease (AOR = 1.71, 95% CI = 1.21–2.42) or hypertension (AOR = 2.69, 95% CI = 2.13–3.40). These findings align with those of our primary analysis of the impact of DSR when individuals with chronic kidney disease or hypertension treated by a doctor in 1999 were excluded from the analysis. To establish causality, future studies should focus on identifying reliable instruments for determining the need for DSR.

Previous research has consistently demonstrated a higher prevalence of frailty among older women compared with older men [46,47,48]. This study examined this aspect by conducting a subgroup analysis and found that both female and male older adults were susceptible to developing frailty if DSR was recommended. Older adults without a formal education also had a higher likelihood of reporting frailty [49,50,51,52]. We further confirmed that the association between DSR and frailty likelihood was dependent on educational attainment, with no formal education associated with a higher likelihood of frailty. This connection may be explained by the close association between educational level and health literacy [53,54,55]. Despite the recommendation to reduce sodium intake in older adults with higher health literacy, they have the option to engage in other healthy behaviors or adopt alternative diets to avoid the possibility of experiencing prolonged decreased appetite or appetite loss resulting from a low-sodium diet. This claim, however, requires further evidence.

There was an age-dependent effects of how DSR was linked to frailty. DSR had a smaller impact on frailty for very old adults (80 years and above). We speculated that the very old population exhibits a significant likelihood of frailty due to the age-dependent declines in the functioning of multiple organ systems, thereby diminishing the impact of DSR on frailty. Existing research indicates that frailty is more prevalent with increasing age [56,57,58]. Further empirical research is required to support this hypothesis.

A temporal pattern was observed in the impact of DSR on the likelihood of frailty. One potential explanation for this is that individuals with DSR are more prone to frailty and mortality than their non-DSR counterparts. The delayed effect of the DSR on the likelihood of frailty and death, observed in the mid-period cohort, indicates an increased risk over time, as confirmed by our findings (Figure S3). Thus, the insignificant long-term effects of DSR may be attributed to the inadvertent absence of follow-up due to mortality. However, additional evidence is crucial to support this hypothesis.

This study has several limitations that warrant further examination. First, there was only a baseline assessment of the DSR; any subsequent changes may have resulted in or been influenced by physical changes that occurred throughout the follow-up period. However, in contrast to self-reported nutritional status, sodium restriction is frequently prescribed by physicians to maintain control of essential hypertension and kidney disease. Second, the TLSA did not include objective data regarding the dietary salt intake level based on body physique, such as height and weight, for participants being ordered to DSR by a physician. Furthermore, the TLSA lacked sufficient information to ascertain whether participants fully comprehended the DSR. Future research that scrutinizes these issues through urine collection would help us understand the recommended daily salt intake. Third, patients took diuretics that caused some form of frailty. Specific medication data were not included in the TLSA. However, conditions such as heart disease, hypertension, and chronic kidney disease that may lead to edema or necessitate diuretics were taken into account when including comorbidity as a control variable. Furthermore, participants with hypertension and chronic kidney disease were excluded from this study because frailty may be caused by the use of diuretics rather than the DSR. The results remained as expected. Fourth, a potential link was observed between the DSR and frailty in older adults. However, a deeper exploration using biomarker data could shed light on the mechanisms through which dietary salt restriction induces physiological and anatomical changes, negatively affecting health status. Finally, environmental-level factors (e.g., housing, facilities, neighborhood, noise, and traffic) may introduce bias in our results. Hence, future studies should explore the cross-level interactions. However, this improvement in the living environment design could also be a consequence of frailty, and caution must be exercised when interpreting the endogenous relationships between environmental variables and frailty.

## 5. Conclusions

Our study revealed the significance of DSR for addressing frailty-related concerns in later life. At least three of these implications warrant further investigation. First, efforts to decrease the likelihood of frailty among middle-aged and older adults should begin with a focus on DSR. Second, reducing the negative impact of DSR on frailty might be more effective if the use of intervention strategies is targeted at older adults with no formal education or in short- and mid-period cohorts. Third, improving nutritional and functional status is a top priority for anyone who needs to reduce their dietary sodium intake, as DSR is associated with a significantly increased risk of SBF.

## Figures and Tables

**Table 1:** Characteristics of a sample of pooled cross-sectional and time-series observations: frailty status defined by SOF frailty index.

	Frailty (SOF)		*p*-Value
	No	Yes	
N	7539	818	
(%)	(90.21)	(9.79)	
Age			<0.001
50–64	2466	86	
	(32.71)	(10.51)	
65–79	4106	476	
	(54.46)	(58.19)	
≥80	967	256	
	(12.83)	(31.30)	
Age (continuous)			<0.001
Sex			<0.001
Female	3285	515	
	(43.57)	(62.96)	
Male	4254	303	
	(56.43)	(37.04)	
Education (years)			<0.001
0	2318	411	
	(30.75)	(50.24)	
1–6	3205	290	
	(42.51)	(35.45)	
7–12	1483	87	
	(19.67)	(10.64)	
≥13	533	30	
	(7.07)	(3.67)	
Marital status ^a^			<0.001
Having a spouse	5296	432	
	(70.25)	(52.81)	
Not having a spouse	2243	386	
	(29.75)	(47.19)	
Current living status			0.413
Living with spouse, child, etc.	6803	731	
	(90.26)	(89.36)	
Living alone	734	87	
	(9.74)	(10.64)	
Smoking status			<0.001
Non-smoker	5784	730	
	(76.72)	(89.24)	
Smoker	1755	88	
	(23.28)	(10.76)	
Alcohol intake			<0.001
Non-alcohol drinker	5410	734	
	(71.76)	(89.73)	
Alcohol drinker	2129	84	
	(28.24)	(10.27)	
Frequency of exercise (times per week)			<0.001
0	2475	449	
	(32.83)	(54.89)	
1	481	33	
	(6.38)	(4.03)	
2	860	84	
	(11.41)	(10.27)	
≥3	3722	252	
	(49.38)	(30.81)	
Dietary sodium restriction			<0.001
No	6436	614	
	(86.66)	(76.37)	
Yes	991	190	
	(13.34)	(23.63)	
Year			<0.001
1999	3336	257	
	(44.25)	(31.42)	
2003	2362	307	
	(31.33)	(37.53)	
2007	1841	254	
	(24.42)	(31.05)	

Note: missing values in some variables in the dataset. ^a^ Having a spouse: Living with a partner or married; Not having a spouse: Never married/separated/divorced/widowed.

## Data Availability

The data presented in this study are available on request from the corresponding author (politicshtyan@gmail.com).

## Supplementary Materials

The following supporting information can be downloaded at: https://www.mdpi.com/article/10.3390/nu16050580/s1, Figure S1: The effects of DSR on the probability of the robust, pre-frailty, and frailty group among middle-aged and older adults, Taiwan, 1999–2007. Figure S2: The temporal effects of DSR on the probability of the robust, pre-frailty, and frailty group among middle-aged and older adults, Taiwan, 1999–2007. Figure S3: The temporal effects of DSR on the probability of death after frailty among middle-aged and older adults, Taiwan, 1999–2007. Table S1: Robustness check: predictors of dietary sodium restriction that may influence frailty among older adults in Taiwan, 1999–2007.
