# Supplementary material for: Dietary Sodium Restriction and Frailty among Middle-Aged and Older Adults: An 8-Year Longitudinal Study
Source: Nutrients. 2024 Feb 20;16(5):580. doi: 10.3390/nu16050580 (PMC10933923; doi:10.3390/nu16050580)
Supplement: Supplementary file 1 [file nutrients-16-00580-s001.zip › nutrients-2856808-supplementary.pdf]

Supplementary information

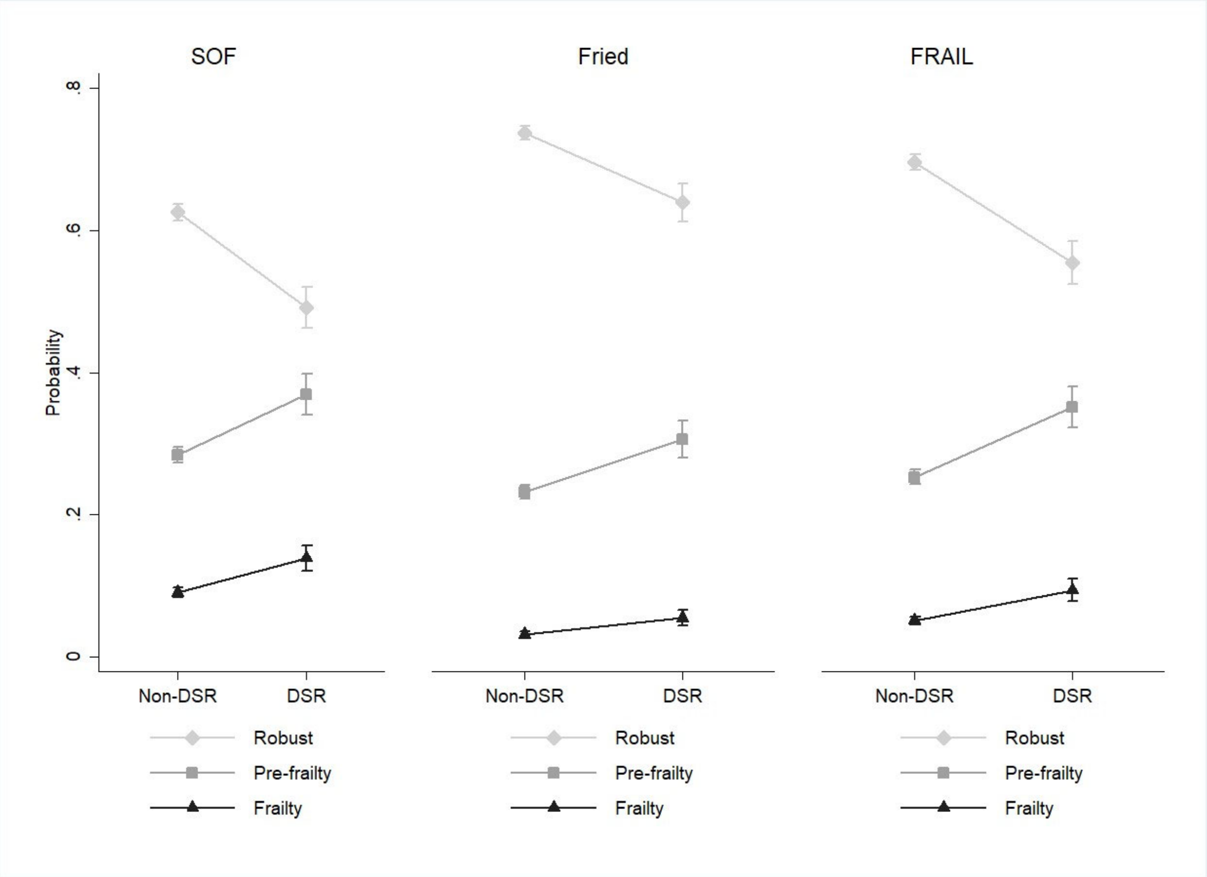

Figure S1. The effects of DSR on the probability of the robust, pre-frailty, and frailty group among middle-aged and older adults, Taiwan, 1999–2007

Note: All results were based on random-effects panel logit model. The data points represent the mean ± standard error. AOR: adjusted odds ratio; DSR: dietary sodium restriction. Probability(pre-frailty) (SOF): DSR: 0.37^{***}, CI = 0.34–0.40; Non-DSR: 0.28^{***}, CI = 0.27–0.29; Probability(pre-frailty) (Fried): DSR: 0.31^{***}, CI = 0.28–0.33; Non-DSR: 0.23^{***}, CI = 0.22–0.24; Probability(pre-frailty) (FRAIL): DSR: 0.35^{***}, CI = 0.32–0.38; Non-DSR: 0.25^{***}, CI = 0.24–0.26; * $p < 0.05$, ** $p < 0.01$, and *** $p < 0.001$. *Source*: the author.

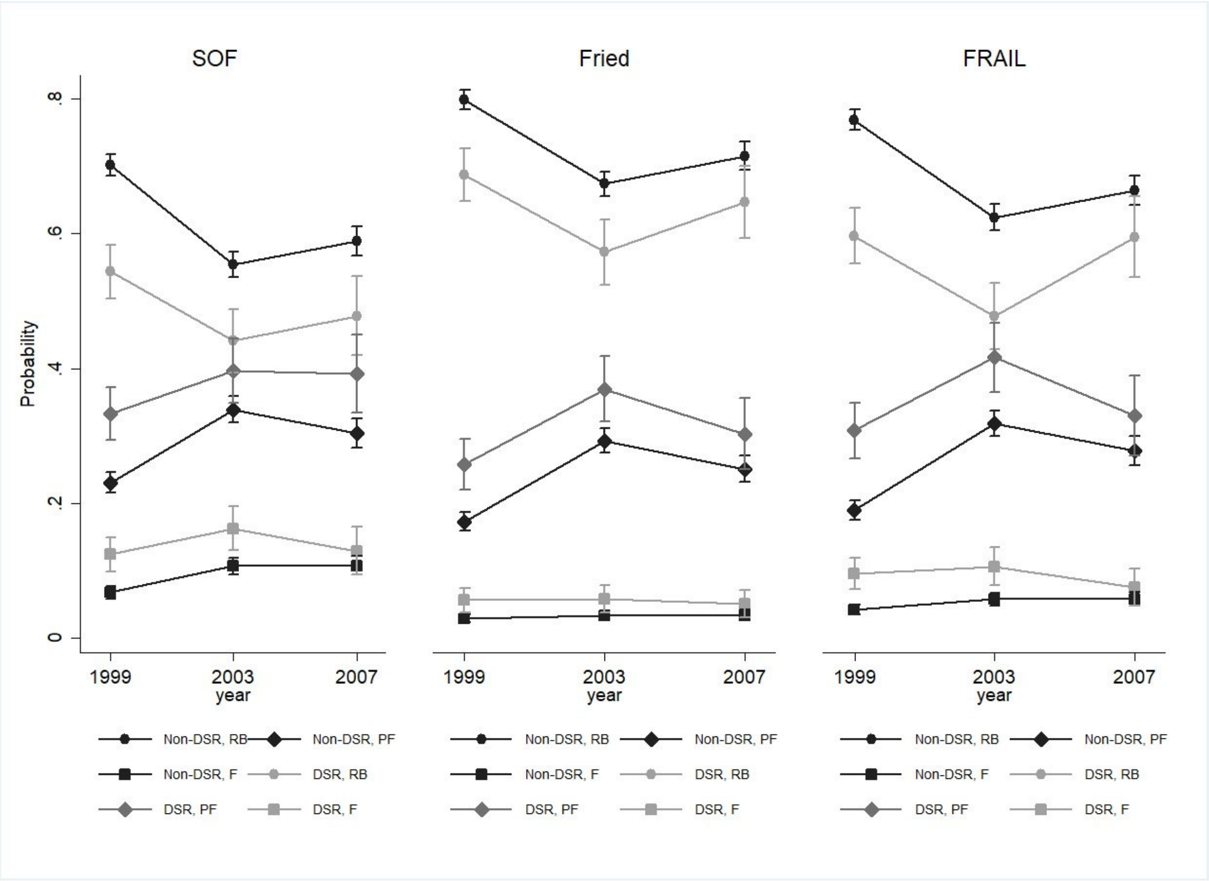

Figure S2. The temporal effects of DSR on the probability of the robust, pre-frailty, and frailty group among middle-aged and older adults, Taiwan, 1999–2007

Note: All results were based on random-effects panel logit model. The data points represent the mean ± standard error. AOR: adjusted odds ratio; DSR: dietary sodium restriction. Probability(pre-frailty) (SOF): DSR, 1999: 0.33***, CI = 0.29–0.37; Non-DSR, 1999: 0.23***, CI = 0.22–0.25; DSR, 2003: 0.40***, CI = 0.35–0.44; Non-DSR, 2003: 0.34***, CI = 0.32–0.36; DSR, 2007: 0.39***, CI = 0.33–0.45; Non-DSR, 2007: 0.30***, CI = 0.28–0.33; Probability(pre-frailty) (Fried): DSR, 1999: 0.26***, CI = 0.22–0.30; Non-DSR, 1999: 0.17***, CI = 0.16–0.19; DSR, 2003: 0.37***, CI = 0.32–0.42; Non-DSR, 2003: 0.29***, CI = 0.27–0.31; DSR, 2007: 0.30***, CI = 0.25–0.36; Non-DSR, 2007: 0.25***, CI = 0.23–0.27; Probability(pre-frailty) (FRAIL): DSR, 1999: 0.31***, CI = 0.27–0.35; Non-DSR, 1999: 0.19***, CI = 0.17–0.20; DSR, 2003: 0.42***, CI = 0.37–0.47; Non-DSR, 2003: 0.32***, CI = 0.30–0.34; DSR, 2007: 0.33***, CI = 0.27–0.39; Non-DSR, 2007: 0.28***, CI = 0.26–0.30; * $p < 0.05$, ** $p < 0.01$, and *** $p < 0.001$. *Source*: the author.

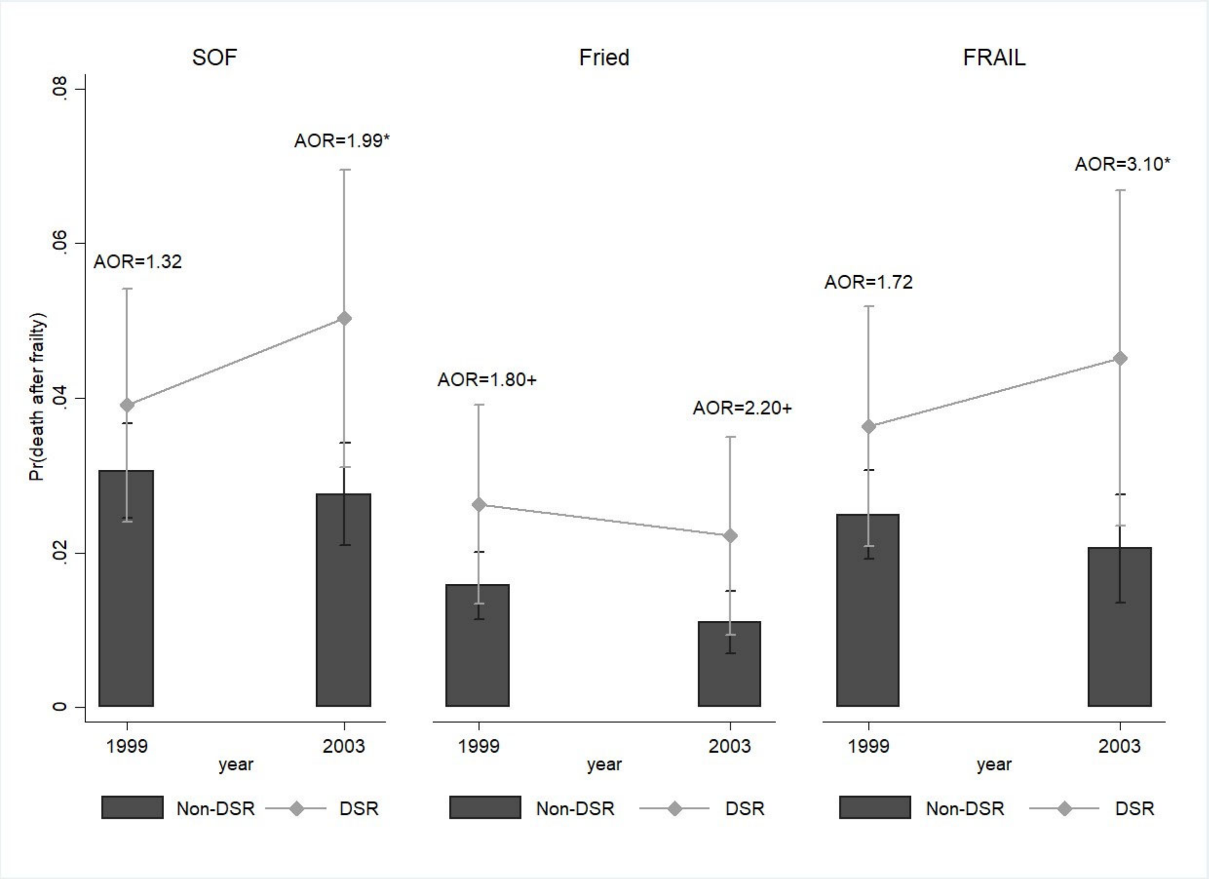

Figure S3. The temporal effects of DSR on the probability of death after frailty among middle-aged and older adults, Taiwan, 1999–2007

Note: All results were based on random-effects panel logit model. The data points represent the mean ± standard error. Pr(death after frailty): the predicted probability of frailty; AOR: adjusted odds ratio; DSR: dietary sodium restriction. Pr(death after frailty) (SOF): DSR, 1999–2003: 0.04^{***}, CI = 0.02–0.05; Non-DSR, 1999–2003: 0.03^{***}, CI = 0.02–0.04; DSR, 2003–2007: 0.05^{***}, CI = 0.03–0.07; Non-DSR, 2003–2007: 0.03^{***}, CI = 0.02–0.03; Pr(death after frailty) (Fried): DSR, 1999–2003: 0.03^{***}, CI = 0.01–0.04; Non-DSR, 1999–2003: 0.02^{***}, CI = 0.01–0.02; DSR, 2003–2007: 0.02^{***}, CI = 0.01–0.03; Non-DSR, 2003–2007: 0.01^{***}, CI = 0.01–0.02; Pr(death after frailty) (FRAIL): DSR, 1999–2003: 0.04^{***}, CI = 0.02–0.05; Non-DSR, 1999–2003: 0.02^{***}, CI = 0.02–0.03; DSR, 2003–2007: 0.05^{***}, CI = 0.02–0.07; Non-DSR, 2003–2007: 0.02^{***}, CI = 0.01–0.03; $^{+}p < 0.1$, $^{*}p < 0.05$, $^{**}p < 0.01$, and $^{***}p < 0.001$. *Source*: the author.

Tables

Table S1. Robustness check: predictors of dietary sodium restriction that may influence frailty among older adults in Taiwan, 1999–2007

	Model 1[a]	Model 2[b]	Model 3[c]	Model 4[a]	Model 5[b]
	Frailty (SOF) (reference="no")	Frailty (SOF) (reference="no")	Frailty (SOF) (reference="no")	Frailty (Fried) (reference="no")	Frailty (Fried) (reference="no")
	Yes	Yes	Yes	Yes	Yes
	AOR (95% CI)	AOR (95% CI)	AOR (95% CI)	AOR (95% CI)	AOR (95% CI)
Dietary Sodium Restriction (DSR)					
No	REF	REF	REF	REF	REF
Yes	2.59 (2.05 , 3.28)***[d]	2.13 (1.47 , 3.10)***	1.73 (1.38 , 2.18)***	2.37 (1.75 , 3.22)***	3.07 (1.38 , 6.80)**
DSR × Year 1999					
1999, Non-DSR	REF	REF	REF	REF	REF
1999, DSR	3.29 (2.32 , 4.66)***	2.68 (1.53 , 4.70)***	2.20 (1.54 , 3.14)***	2.77 (1.71 , 4.49)***	3.99 (1.37 , 11.61)*
DSR × Year 2003					
2003, Non-DSR	REF	REF	REF	REF	REF
2003, DSR	2.42 (1.67 , 3.51)***	2.83 (1.64 , 4.90)***	1.73 (1.21 , 2.48)**	2.37 (1.41 , 4.00)**	3.09 (0.94 , 10.22)+
DSR × Year 2007					
2007, Non-DSR	REF	REF	REF	REF	REF
2007, DSR	1.81 (1.15 , 2.86)*	0.82 (0.36 , 1.88)	1.24 (0.80 , 1.91)	1.86 (1.04 , 3.32)*	1.87 (0.46 , 7.59)

Note: AOR, adjusted odds ratio; CI, confidence interval; REF, reference groups. Individual-level characteristics were set as control variables. These included gender, age, education level, marital status, current living status, smoking status, alcohol intake, and frequency of exercise. [a] The outcome variable was categorized into three distinct three different groups. [b] Individuals were excluded from analysis if they were diagnosed with hypertension and treated (or medicated) by a doctor in 1999. [c] Individuals were excluded from analysis if they were diagnosed with chronic kidney disease and treated (or medicated) by a doctor in 1999. [d] + $p < 0.1$, * $p < 0.05$, ** $p < 0.01$, and *** $p < 0.001$.

Table S1. Robustness check: continue

	Model 6[c]	Model 7[a]	Model 8[b]	Model 9[c]	Model 10[d]
	Frailty (Fried) (reference="no")	Frailty (FRAIL) (reference="no")	Frailty (FRAIL) (reference="no")	Frailty (FRAIL) (reference="no")	SBF (reference="no")
	Yes	Yes	Yes	Yes	Yes
	AOR (95% CI)	AOR (95% CI)	AOR (95% CI)	AOR (95% CI)	AOR (95% CI)
Dietary Sodium Restriction (DSR)					
No	REF	REF	REF	REF	REF
Yes	2.50 (1.57 , 3.98)***e	3.14 (2.39 , 4.13)***	2.51 (1.38 , 4.59)**	2.47 (1.72 , 3.56)***	2.70 (1.63 , 4.48)***
DSR × Year 1999					
1999, Non-DSR	REF	REF	REF	REF	REF
1999, DSR	2.61 (1.34 , 5.07)**	4.33 (2.86 , 6.54)***	4.06 (1.74 , 9.47)**	3.06 (1.81 , 5.18)***	2.19 (1.08 , 4.42)*
DSR × Year 2003					
2003, Non-DSR	REF	REF	REF	REF	REF
2003, DSR	2.81 (1.38 , 5.70)**	3.26 (2.07 , 5.13)***	2.71 (1.09 , 6.73)*	2.80 (1.61 , 4.85)***	4.22 (2.00 , 8.90)***
DSR × Year 2007					
2007, Non-DSR	REF	REF	REF	REF	REF
2007, DSR	2.03 (0.92 , 4.49)+	1.65 (0.95 , 2.87)+	0.96 (0.29 , 3.14)	1.43 (0.73 , 2.81)	1.94 (0.76 , 4.93)

Note: AOR, adjusted odds ratio; CI, confidence interval; REF, reference groups. Individual-level characteristics were set as control variables. These included gender, age, education level, marital status, current living status, smoking status, alcohol intake, and frequency of exercise. [a] The outcome variable was categorized into three distinct three different groups. [b] Individuals were excluded from analysis if they were diagnosed with hypertension and treated (or medicated) by a doctor in 1999. [c] Individuals were excluded from analysis if they were diagnosed with chronic kidney disease and treated (or medicated) by a doctor in 1999. [d] The study used an alternative measure to lower limb muscle strength, which is one component of the SBF scale. [e] + $p < 0.1$, * $p < 0.05$, ** $p < 0.01$, and *** $p < 0.001$.
